# Supplementary material for: Genes associated with cognitive ability and HAR show overlapping expression patterns in human cortical neuron types
Source: Nat Commun. 2023 Jul 13;14:4188. doi: 10.1038/s41467-023-39946-9 (PMC10345092; doi:10.1038/s41467-023-39946-9)
Supplement: Supplementary file 1 — Supplementary Information [file 41467_2023_39946_MOESM1_ESM.pdf]

Supplementary information

Genes associated with cognitive ability and HAR show overlapping expression patterns in human cortical neuron types

Stan LW Driessens [1], Anna A Galakhova [1], Djai B Heyer [1], Isabel J Pieterse[1], René Wilbers [1], Eline J Mertens [1], Femke Waleboer [1], Tim S Heistek [1], Loet Coenen [1], Julia R Meijer [1], Sander Idema [2], Philip C de Witt Hamer [2], David P Noske [2], Christiaan PJ de Kock [1], Brian R Lee [3], Kimberly Smith [3], Jonathan T Ting [3], Ed S Lein [3], Huibert D Mansvelder [1], Natalia A Goriounova [1]*

Supplementary Table 1

Gene Name	Annotation	Associated disease
**ADCY8**	adenylate cyclase 8	Dissociative Amnesia;<br>Joubert Syndrome 24
ADTRP	Androgen Dependent TFPI Regulating Protein	Nonobstructive Coronary Artery Disease
AKAP6	A-Kinase Anchoring Protein 6	Ventricular Tachycardia
CACNA2D3	Calcium Voltage-Gated Channel Auxiliary Subunit Alpha2delta 3	Frontonasal Dysplasia 1;<br>Alkuraya-Kucinskas Syndrome
**CADPS2**	calcium dependent secretion activator 2	Autism;<br>Intellectual Developmental Disorder
CDH20	cadherin 20	Craniofacial-Deafness-Hand Syndrome;<br>Choanal Atresia, Posterior
**CDH8**	cadherin 8	Craniofacial-Deafness-Hand Syndrome;<br>Ectodermal Dysplasia;<br>Ectrodactyly;<br>Macular Dystrophy Syndrome
COL11A1	collagen type XI alpha 1 chain	Stickler Syndrome;<br>Marshall Syndrome
CSMD1	CUB And Sushi Multiple Domains 1	Schizophrenia;<br>Aceruloplasminemia
DAB1	DAB Adaptor Protein 1	Spinocerebellar Ataxia;<br>Spastic Ataxia
DCAF4	DDB1 And CUL4 Associated Factor 4	Microphthalmia;<br>Atrial Septal Defect
DGKG	Diacylglycerol Kinase Gamma	Spinocerebellar Ataxia
**DNM3**	dynamin 3	Autosomal Dominant Optic Atrophy Plus Syndrome;<br>Hereditary Sensory Neuropathy
DPP10	Dipeptidyl Peptidase Like 10	Schizophrenia;<br>Asthma
**EFNA5**	ephrin A5	Cortical Senile Cataract;<br>Persistent Hyperplastic Primary Vitreous
**ELAVL2**	ELAV like RNA binding protein 2	Hyperinsulinemic Hypoglycemia; Myotonic Disease
ETV1	ETS Variant Transcription Factor 1	Ewing Sarcoma;<br>Gastrointestinal Stromal Tumor
GLIS3	GLIS Family Zinc Finger 3	Diabetes Mellitus;<br>Neonatal Diabetes

**GRM3**	glutamate metabotropic receptor 3	Bipolar Disorder;<br>Schizophrenia.
**GRM8**	glutamate metabotropic receptor 8	Schizophrenia;<br>Epilepsy
HS3ST4	Heparan Sulfate-Glucosamine 3-Sulfotransferase 4	Herpes Simplex;<br>Herpes Zoster
**HTR2A**	5-hydroxytryptamine receptor 2A	Major Depressive Disorder;<br>Obsessive-Compulsive Disorder
KCNH5	Potassium Voltage-Gated Channel Subfamily H Member 5	Developmental And Epileptic Encephalopathy;<br>Ohtahara Syndrome;
KCNIP2	Potassium Voltage-Gated Channel Interacting Protein 2	Spinocerebellar Ataxia;<br>Brugada Syndrome
LINGO2	Leucine Rich Repeat And Ig Domain Containing 2	Essential Tremor
LPP	LIM Domain Containing Preferred Translocation Partner In Lipoma	Leukemia, Acute Myeloid;<br>Acute Monoblastic Leukemia.
MAST4	Microtubule Associated Serine/Threonine Kinase Family Member 4	Spinocerebellar Ataxia 27
MBNL2	Muscleblind Like Splicing Regulator 2	Myotonic Dystrophy 1;<br>Myotonic Disease.
MEGF9	Multiple EGF Like Domains 9	Fiedler's Myocarditis;<br>Myopathy;<br>Areflexia;<br>Respiratory Distress;<br>Dysphagia, Early-Onset
MEIS2	Meis Homeobox 2	Mental Retardation;<br>Cleft Palate;<br>Cardiac Defects;<br>Chromosome 15Q14 Deletion Syndrome
NPAS3	Neuronal PAS Domain Protein 3	Schizophrenia
**NSF**	N-ethylmaleimide sensitive factor, vesicle fusing ATPase	Developmental And Epileptic Encephalopathy 96;<br>Tetanus
**NTRK3**	neurotrophic receptor tyrosine kinase 3	Congenital Mesoblastic Nephroma;<br>Glioma
NYAP2	Neuronal Tyrosine-Phosphorylated Phosphoinositide-3-Kinase Adaptor 2	Astigmatism
OLFM3	Olfactomedin 2	Renal Pelvis Carcinoma;<br>Glaucoma, Primary Open Angle
OPCML	Opioid Binding Protein/Cell Adhesion Molecule Like	Ovarian Cancer;<br>Hypogonadotropic Hypogonadism 14 With/Or Without Anosmia
PBRM1	Polybromo 1	Renal Cell Carcinoma;<br>Nonpapillary and Clear Cell Papillary Renal Cell Carcinoma
PITPNC1	Phosphatidylinositol Transfer Protein Cytoplasmic 1	Retinal Degeneration
POU6F2	POU Class 6 Homeobox 2	Wilms Tumor 5;<br>Wilms Tumor 1
**PTPRD**	protein tyrosine phosphatase receptor type D	Restless Legs Syndrome;<br>Chromosome 9P Deletion Syndrome

**PTPRT**	protein tyrosine phosphatase receptor type T	Autism;<br>Schizophrenia
**RIMS1**	regulating synaptic membrane exocytosis 1	Cone-Rod Dystrophy 7;<br>Gyrate Atrophy of Choroid and Retina
RORA	RAR Related Orphan Receptor A	Intellectual Developmental Disorder With/Or Without Epilepsy or Cerebellar Ataxia; Epidermolysis Bullosa Acquisita
SCAPER	S-Phase Cyclin A Associated Protein In The ER	Intellectual Developmental Disorder and Retinitis Pigmentosa;<br>Speech Disorder
SEMA6A	Semaphorin 6A	Deafness, Autosomal Recessive 97;<br>Exfoliation Syndrome
SEMA6D	Semaphorin 6D	Cone-Rod Dystrophy 10;<br>Inflammatory Bowel Disease
SGCZ	Sarcoglycan Zeta	Hallucinogen Abuse;<br>Dystonia 11, Myoclonic
SND1	Staphylococcal Nuclease And Tudor Domain Containing 1	Posterior Pituitary Gland Neoplasm;<br>Pancreatic Acinar Cell Adenocarcinoma
**SORCS3**	sortilin related VPS10 domain containing receptor 3	Attention Deficit-Hyperactivity Disorder
SOX5	SRY-Box Transcription Factor 5	Lamb-Shaffer Syndrome;<br>Optic Nerve Hypoplasia, Bilateral
SPRY4	Sprouty RTK Signaling Antagonist 4	Hypogonadotropic Hypogonadism 17 With/Or Without Anosmia;<br>Kallmann Syndrome.
STXBP6	Syntaxin Binding Protein 6	Deafness, Autosomal Recessive 35; Syndromic X-Linked Intellectual Disability Cabezas Type
TCERG1L	Transcription Elongation Regulator 1 Like	-
TMTC2	Transmembrane O-Mannosyltransferase Targeting Cadherins 2	Epilepsy, Familial Temporal Lobe, 5;<br>Arteritic Anterior Ischemic Optic Neuropathy
TRIB2	Tribbles Pseudokinase 2	-
TROVE2	Ro60, Y RNA Binding Protein	Sjogren Syndrome;<br>Lupus Erythematosus.
TRPM3	Transient Receptor Potential Cation Channel Subfamily M Member 3	Autosomal Dominant Non-Syndromic Intellectual Disability;<br>Mulibrey Nanism
TSHZ3	Teashirt Zinc Finger Homeobox 3	Hydronephrosis;<br>Prune Belly Syndrome
VPS41	VPS41 Subunit Of HOPS Complex	Spinocerebellar Ataxia, Autosomal Recessive 929 and 4)
ZEB2	Zinc Finger E-Box Binding Homeobox 2	Mowat-Wilson Syndrome;<br>Nervous System Disease
ZNF521	Zinc Finger Protein 521	Invasive Bladder Transitional Cell Carcinoma;<br>Gastrointestinal Neuroendocrine Benign Tumor
ZNF608	Zinc Finger Protein 608	Leukodystrophy, Demyelinating, Adult-Onset, Autosomal Dominant

Table S1. Gene names and their annotations from the gene set of interest (genes from HAR, IQ, EA associated gene sets that correlated to TDL or AP and belonged to at least two gene sets). Gene names in bold were overrepresented in synaptic process or structure in SynGo analysis (Figure 5 E, F, G). Function and disease annotation of these genes was performed using GeneCards – the human gene database [1,2].

Supplementary Fig 1

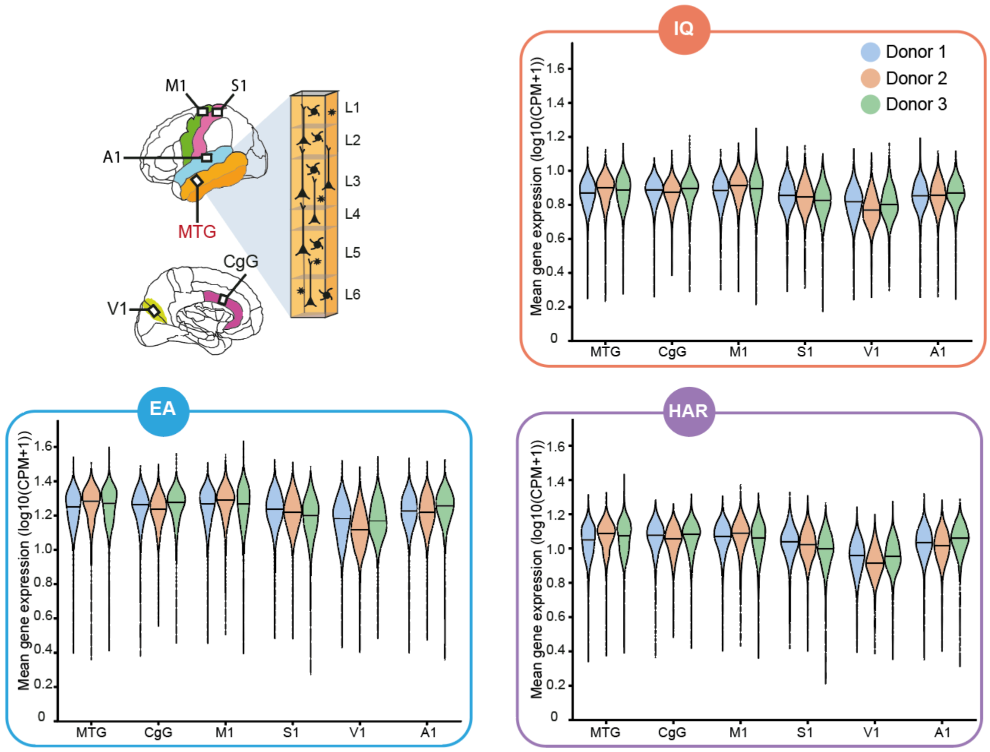

Supplementary Figure 1. Comparisons of cellular gene expression within donor show similar results as pooled data from Figure 1: between donor variance is 100-fold lower than within donor variance. Violins represents the distributions of cellular gene expression data from each donor across brain areas, same data as in Figure 1. The horizontal lines are median values. K-W results: IQ donor 1: p=1.74 x 10^{-168}, donor 2 p=0, donor 3 p=0; EA donor 1 p= 3.6 x 10^{-174}, donor 2 p=0, donor 3 p=6.3 x 10^{-298}; HAR donor 1 p=0, donor 2 p=0, donor 3 p=0. IQ gene set: variance within donors: donor1=0.024, donor2=0.027, donor3=0.025; variance between donors=0.00025. EA gene set: variance within donors: donor1=0.045, donor2=0.053, donor3=0.048; variance between donors=0.00054. HAR gene set: variance within donors: donor1=0.029, donor2=0.033, donor3=0.03; variance between donors=0.00047.

Supplementary Fig 2

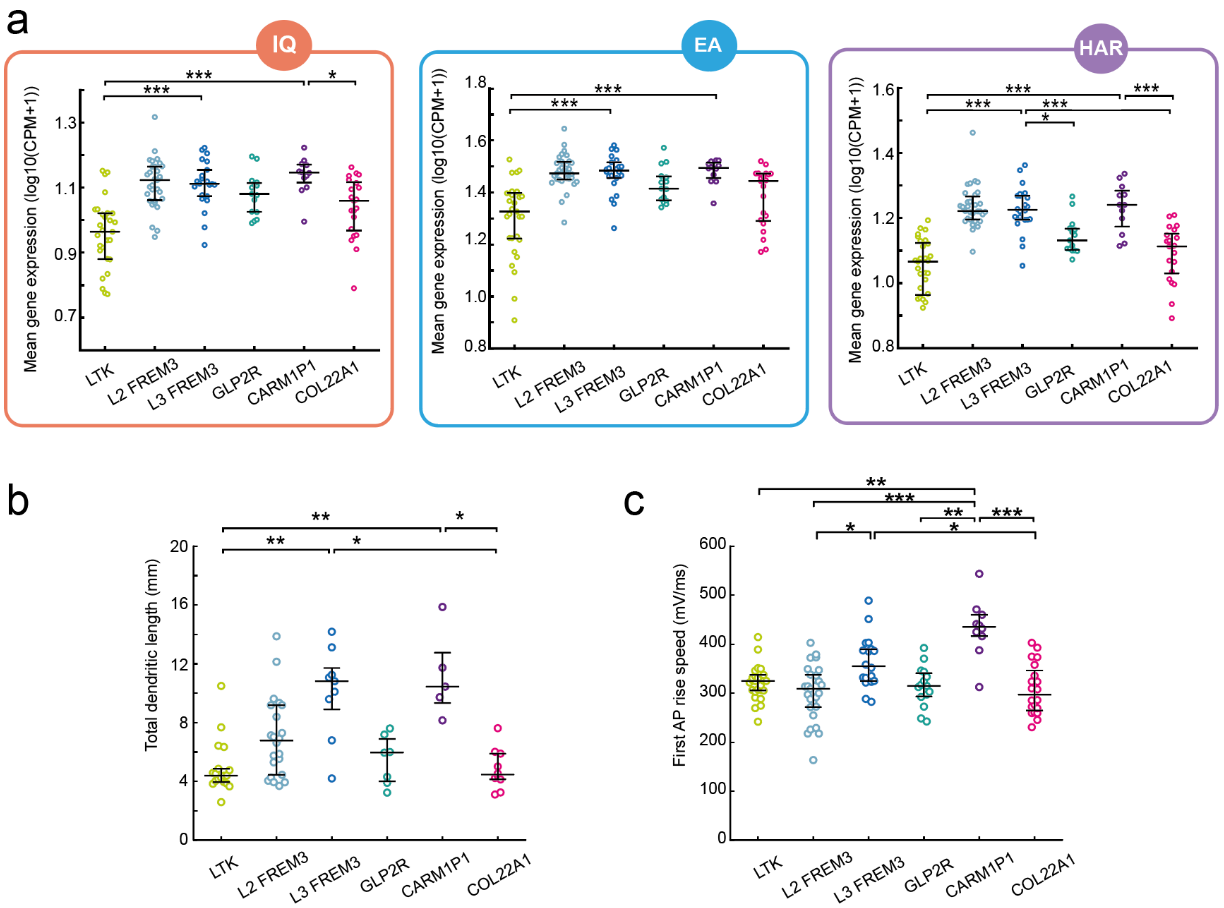

Supplementary Figure 2. Gene expression levels, TDL and AP rise speed show similar results when averaged per donor. (A) Patch-seq data (as in from Figure 2C) displayed as average expression levels of IQ, EA and HAR genes per donor. Kruskal-Wallis (KW) test; IQ gene set: p=1.9 x 10^{-8}; EA gene set: p=3.5 x 10^{-8}; HAR gene set: p=7.2 x 10^{-15}; N (donors) for each group: LTK=29; L2 FREM3=31; L3 FREM3=22; GLP2R=15; CARM1P1=12; COL22A1=21. (B) TDL and (C) AP rise speed in patch-seq cells were averaged per donor for each cell type. TDL data: KW test: p= 1.9 x 10^{-8}; N (donors): LTK=18; L2 FREM3=22; L3 FREM3=9; GLP2R=7; CARM1P1=5; COL22A1=10. AP data: KW test: p= 4.6 x 10^{-6}; N (donors): LTK=26; L2 FREM3=25; L3 FREM3=18; GLP2R=15; CARM1P1=10; COL22A1=20. Asterisks represent p-values of post-hoc comparisons for only CARM1P1 and L3 FREM3 types: *p<0.05; **p<0.01; ***p<0.001. Plots indicate median (middle line) and 25th and 75th percentiles (whiskers).

Supplementary References

1. GeneCards – the human gene database. Available from www.genecards.org.

2. Safran, M. *et al.* The GeneCards Suite. in *Practical Guide to Life Science Databases* 27–56 (Springer Nature Singapore, 2021). doi:10.1007/978-981-16-5812-9_2.
