# Supplementary material for: Genes associated with cognitive ability and HAR show overlapping expression patterns in human cortical neuron types
Source: Nat Commun. 2023 Jul 13;14:4188. doi: 10.1038/s41467-023-39946-9 (PMC10345092; doi:10.1038/s41467-023-39946-9)
Supplement: Supplementary file 3 — Reporting Summary [file 41467_2023_39946_MOESM3_ESM.pdf]

nature portfolio

Corresponding author(s): N.A. Goriounova

Last updated by author(s): 05-06-2023

Reporting Summary

Nature Portfolio wishes to improve the reproducibility of the work that we publish. This form provides structure for consistency and transparency in reporting. For further information on Nature Portfolio policies, see our Editorial Policies and the Editorial Policy Checklist.

Statistics

For all statistical analyses, confirm that the following items are present in the figure legend, table legend, main text, or Methods section.

n/a	Confirmed	
☐	☒	The exact sample size (*n*) for each experimental group/condition, given as a discrete number and unit of measurement
☒	☐	A statement on whether measurements were taken from distinct samples or whether the same sample was measured repeatedly
☐	☒	The statistical test(s) used AND whether they are one- or two-sided<br>*Only common tests should be described solely by name; describe more complex techniques in the Methods section.*
☒	☐	A description of all covariates tested
☐	☒	A description of any assumptions or corrections, such as tests of normality and adjustment for multiple comparisons
☐	☒	A full description of the statistical parameters including central tendency (e.g. means) or other basic estimates (e.g. regression coefficient) AND variation (e.g. standard deviation) or associated estimates of uncertainty (e.g. confidence intervals)
☐	☒	For null hypothesis testing, the test statistic (e.g. *F*, *t*, *r*) with confidence intervals, effect sizes, degrees of freedom and *P* value noted<br>*Give P values as exact values whenever suitable.*
☒	☐	For Bayesian analysis, information on the choice of priors and Markov chain Monte Carlo settings
☒	☐	For hierarchical and complex designs, identification of the appropriate level for tests and full reporting of outcomes
☒	☐	Estimates of effect sizes (e.g. Cohen's *d*, Pearson's *r*), indicating how they were calculated

Our web collection on statistics for biologists contains articles on many of the points above.

Software and code

Policy information about availability of computer code

Data collection	no code was used for data collection
Data analysis	All custom-made script used for the analysis will be provided upon publication

For manuscripts utilizing custom algorithms or software that are central to the research but not yet described in published literature, software must be made available to editors and reviewers. We strongly encourage code deposition in a community repository (e.g. GitHub). See the Nature Portfolio guidelines for submitting code & software for further information.

Data

Policy information about availability of data

All manuscripts must include a data availability statement. This statement should provide the following information, where applicable:

- Accession codes, unique identifiers, or web links for publicly available datasets
- A description of any restrictions on data availability
- For clinical datasets or third party data, please ensure that the statement adheres to our policy

The snRNA-sequencing data used for the analysis in Figure 1, 2 and Figure S1 were obtained from publicly available database at https://celltypes.brain-map.org/ as datasets (Allen Institute for Brain Science 2020; 2018). Patch-seq data used for the analyses in Figures 3-5 are publicly available at https://celltypes.brain-map.org/ (Allen Institute for Brain Science 2020). The patch-seq data (CPM values, cell types, TDL and AP) used for the analyses in Figures 3 and 4 are included in a csv file data_cpm.csv, the file and associated scripts for analysis are provided at the repository DataverseNL: https://doi.org/10.34894/YZFQAO. GO data used for the

analysis in Figure 5 were obtained from MSigDB database available at https://www.gsea-msigdb.org/gsea/msigdb. Overrepresentation analysis of synaptic genes in Figure 5 was performed using the open-source knowledgebase SynGo available at https://www.syngoportal.org. All data in the figures, N numbers, the results of statistical analyses and post-hoc tests are reported in the Source Data file and provided with this paper.

Human research participants

Policy information about studies involving human research participants and Sex and Gender in Research.

Reporting on sex and gender	The cellular RNA-sequencing data were obtained from publicly available databases at https://celltypes.brain-map.org/ (see above) and are available at https://celltypes.brain-map.org/(Allen Institute for Brain Science 2020). The demographic data are reported in the files accompanying this original database, and not separately in the manuscript.
Population characteristics	age of subjects was 18-70 years old, gender information not available
Recruitment	Subjects were epilepsy or brain tumor patient undergoing brain surgery, before the surgery subjects signed informed consent for the use of resected brain tissue and data for research.
Ethics oversight	The procedures were approved by hospital institute review boards (Harborview Medical Center, Swedish Medical Center and University of Washington Medical Center, Vrije Universiteit Amsterdam Medical Center) before commencing the study and all subjects provided written informed consent for the use of data and tissue for scientific research.

Note that full information on the approval of the study protocol must also be provided in the manuscript.

Field-specific reporting

Please select the one below that is the best fit for your research. If you are not sure, read the appropriate sections before making your selection.

☒ Life sciences ☐ Behavioural & social sciences ☐ Ecological, evolutionary & environmental sciences

For a reference copy of the document with all sections, see nature.com/documents/nr-reporting-summary-flat.pdf

Life sciences study design

All studies must disclose on these points even when the disclosure is negative.

Sample size	snRNA-sequencing data originates from 4 donors, Patch-seq data originates from 57 donors. The sample size was limited by the availability of data.
Data exclusions	All data were included after appropriate quality checks
Replication	All data and scripts are available. We have across different labs and the results obtained in VU Amsterdam lab could replicate the results collected by AIBS.
Randomization	Randomization was not used since the data was collected from only one group of human subjects (neurosurgery patients)
Blinding	All human subject data were anonymized and the researchers were blinded to these data.

Reporting for specific materials, systems and methods

We require information from authors about some types of materials, experimental systems and methods used in many studies. Here, indicate whether each material, system or method listed is relevant to your study. If you are not sure if a list item applies to your research, read the appropriate section before selecting a response.

Materials & experimental systems

n/a	Involved in the study	
☒	☐	Antibodies
☒	☐	Eukaryotic cell lines
☒	☐	Palaeontology and archaeology
☒	☐	Animals and other organisms
☒	☐	Clinical data
☒	☐	Dual use research of concern

Methods

n/a	Involved in the study	
☒	☐	ChIP-seq
☒	☐	Flow cytometry
☒	☐	MRI-based neuroimaging
